# Evaluation of the DREAM Technique for a High-Throughput Deorphanization of Chemosensory Receptors in *Drosophila*

**DOI:** 10.3389/fnmol.2018.00366

**Published:** 2018-10-09

**Authors:** Sarah Koerte, Ian W. Keesey, Mohammed A. Khallaf, Lucas Cortés Llorca, Ewald Grosse-Wilde, Bill S. Hansson, Markus Knaden

**Affiliations:** ^1^Department of Evolutionary Neuroethology, Max Planck Institute for Chemical Ecology, Jena, Germany; ^2^Department for Molecular Ecology, Max Planck Institute for Chemical Ecology, Jena, Germany

**Keywords:** *Drosophila*, olfaction, ligand–receptor interaction, DREAM, deorphanization

## Abstract

In the vinegar fly *Drosophila melanogaster*, the majority of olfactory receptors mediating the detection of volatile chemicals found in their natural habitat have been functionally characterized (deorphanized) *in vivo*. In this process, receptors have been assigned ligands leading to either excitation or inhibition in the olfactory sensory neuron where they are expressed. In other, non-drosophilid insect species, scientists have not yet been able to compile datasets about ligand–receptor interactions anywhere near as extensive as in the model organism *D. melanogaster*, as genetic tools necessary for receptor deorphanization are still missing. Recently, it was discovered that exposure to artificially high concentrations of odorants leads to reliable alterations in mRNA levels of interacting odorant receptors in mammals. Analyzing receptor expression after odorant exposure can, therefore, help to identify ligand–receptor interactions *in vivo* without the need for other genetic tools. Transfer of the same methodology from mice to a small number of receptors in *D. melanogaster* resulted in a similar trend, indicating that odorant exposure induced alterations in mRNA levels are generally applicable for deorphanization of interacting chemosensory receptors. Here, we evaluated the potential of the DREAM (**D**eorphanization of **r**eceptors based on **e**xpression **a**lterations in **m**RNA levels) technique for high-throughput deorphanization of chemosensory receptors in insect species using *D. melanogaster* as a model. We confirmed that in some cases the exposure of a chemosensory receptor to high concentration of its best ligand leads to measureable alterations in mRNA levels. However, unlike in mammals, we found several cases where either confirmed ligands did not induce alterations in mRNA levels of the corresponding chemosensory receptors, or where gene transcript-levels were altered even though there is no evidence for a ligand–receptor interaction. Hence, there are severe limitations to the suitability of the DREAM technique for deorphanization as a general tool to characterize olfactory receptors in insects.

## Introduction

Despite more than two decades of research, the mechanisms by which mammals as well as insects detect a seemingly unlimited amount of odorants with a comparably small set of chemosensory receptors remain elusive up to date. Clearly, a one-to-one relationship between volatile chemicals and chemosensory receptors is not plausible. Thus, the general consensus is that insects as well as mammals need a combinatorial code to allow for a differentiation of the plethora of diverse volatile compounds found in nature (Haverkamp et al., 2018). Here, the identity of an odorant would be defined by a pattern of interactions with various chemosensory receptors. One odorant would interact in an excitatory or inhibitory manner with several receptors, and the same chemosensory receptor may interact with a number of different odorants (Malnic et al., 1999).

The drastic discrepancy between the diversity of airborne chemo-signals and the amount of detecting receptors becomes quite prominent in insects. The adult vinegar fly *Drosophila melanogaster* possesses a repertoire of approximately 44 functional odorant receptors (ORs) including the OR co-receptor ORCO, expressed in the olfactory organs, antennae and maxillary palps, solely for the detection of odorants (Vosshall et al., 2000; Couto et al., 2005). Additionally, the olfactory system of the fly deploys 17 chemosensory receptors belonging to the receptor family of ionotropic receptors (IRs) including four co-receptors for the detection of airborne organic acids, aldehydes, and amines (Stocker, 2001; Yao et al., 2005; Benton et al., 2009; Silbering et al., 2011; Menuz et al., 2014) as well as the two gustatory receptors (GRs) Gr21a and Gr63a for carbon-dioxide sensing (Jones et al., 2007; Kwon et al., 2007). Chemosensory receptors involved in olfaction reside in the dendritic membrane of olfactory sensory neurons (OSNs), which are found in groups of two-to-four and are housed in hair-like structures called sensilla on the antennae or maxillary palps (Vosshall and Stocker, 2007; Su et al., 2009). In *D. melanogaster* four morphologically distinct sensillum types (Stocker, 1994; Yao et al., 2005; Su et al., 2009; Martin et al., 2013; Lin and Potter, 2015) take part in the discrimination of different chemical classes: basiconic, intermediate, and coeloconic sensilla house OSNs for detection of general odorants represented by esters, alcohols, aldehydes, amines and acids, respectively (Hallem and Carlson, 2006), while trichoid sensilla are exclusively mediating the perception of pheromones, which are predominantly long fatty acid chains (Clyne et al., 1997; Kurtovic et al., 2007; Dweck et al., 2015b). Summed up, *D. melanogaster* expresses a set of approximately 62 known chemosensory receptor types total on the antennae and maxillary palps conferring the detection of a multitude of different odorants in nature including odorants for the location of food sources (Zhu et al., 2003; Hallem and Carlson, 2006; Dweck et al., 2016) as well as oviposition sites (Dweck et al., 2013), avoidance of harmful microorganisms (Stensmyr et al., 2012) or natural enemies (Ebrahim et al., 2015), and finally for governing courtship behavior (Clyne et al., 1997; Dweck et al., 2015b). These chemosensory receptors can be categorized into two types of receptors, those which only interact with a small set of ligands or even just one odorant, referred to as narrowly tuned receptors, and those which have a broad spectrum of ligands, characterized as broadly tuned receptors.

For the vinegar fly, most chemosensory receptors involved in olfaction have been assigned a “best ligand,” which is an agonist that already at low doses leads to a strong activity of OSNs expressing this receptor. The identification of a chemosensory receptor’s best excitatory ligand is referred to as deorphanization. The deorphanization of chemosensory receptors playing a role in the olfactory system of *D. melanogaster* has been a time-consuming endeavor and has only been possible thanks to the extensive genetic tools available in this model organism, like “empty neuron” or “decoder” systems. These mutant OSNs are lacking their endogenous receptor gene but instead when Gal4/UAS targeted express a receptor of interest, which can thereby be functionally characterized via Single Sensillum Recordings (SSR; Dobritsa et al., 2003; Hallem and Carlson, 2004, 2006; Yao et al., 2005; Grosjean et al., 2011; Silbering et al., 2011; Ronderos et al., 2014; Gonzalez et al., 2016). However, recent findings suggest that not only the excitation of OSNs via their agonists or best ligands is behaviorally relevant for *Drosophila* but also inhibitory interactions of chemosensory receptors and antagonistic ligands seem to play an important role in the perception of odorants leading to a behavioral output (Cao et al., 2017; MacWilliam et al., 2018). The revelation of this phenomenon indicates a bidirectional code in addition to combinatorial coding allowing for an even greater odor-coding capacity by adding another level of complexity, as the excitation or inhibition of an OSN concurring with the activation or inhibition of a set of OSNs expressing other ORs confers different meanings (Cao et al., 2017).

While in *D. melanogaster* scientists have been able to work on understanding the principles underlying the function of the olfactory system, studies are hampered by the lack of genetic tools available in related non-*melanogaster* flies within the genus Drosophilidae. A great progress has been made in the deorphanization of chemosensory receptors in non-drosophilid insect models in the mosquito *Anopheles gambiae* (Wang et al., 2010) and the moth *Spodoptera littoralis* (De Fouchier et al., 2017), but the methods used (mosquito: ectopic expression of receptors in *Xenopus* oocytes; moth: *Drosophila* empty neuron system) were extremely time-consuming. Deorphanization of chemosensory receptors involved in detecting olfactory signals becomes even more time-consuming in insect species without a sequenced, annotated genome, and without an option for the application of genetic tools. Fortunately, in mammals and potentially also in *Drosophila* the discovery of a correlation between prolonged exposure to high odorant concentrations and regulations in mRNA levels of interacting chemosensory receptors (von der Weid et al., 2015) gave rise to a procedure potentially allowing for chemosensory receptor deorphanization in any species of interest without the requirement of genetic tools such as those available in *D. melanogaster*. Furthermore, this method, which is referred to as DREAM (**D**eorphanization of **r**eceptors based on **e**xpression **a**lterations of **m**RNA levels), has the potential to allow identification of all chemosensory receptors interacting with an odorant in a high-throughput manner instead of a deorphanization of single ligand-receptor pairs at a time.

In the present study, we evaluated the applicability of the DREAM technique for a high-throughput deorphanization of general ORs, pheromone receptors (PRs), and IRs utilizing previously established ligand-receptor combinations in *D. melanogaster*. Using RealTime quantitative polymerase chain reaction (qPCR), we were able to reproduce the described down-regulation of target genes by analyzing ligand–receptor combinations tested in the original study (von der Weid et al., 2015). Subsequently, we evaluated the general suitability of the DREAM technique for deorphanization of broadly and narrowly tuned ORs. Here, we were not able to consistently correlate ligand–receptor interactions with alterations in gene transcript-levels; only three out of six additionally tested ORs showed changed mRNA levels upon prolonged exposure to their best known ligand. Furthermore, we tested the applicability of the DREAM technique for the deorphanization of chemosensory receptor classes besides ORs, monitoring the effect of prolonged odorant exposure on the transcription of IRs. However, after odorant treatment, we did not observe any significant changes in the IR’s gene transcription, implying that the DREAM method may not be useful for deorphanization of IRs. Finally, in order to test whether changes in the experimental conditions of the DREAM technique would lead to more reliable results, we varied odorant exposure duration but did not obtain different results.

In summary, while in certain cases, we confirmed that transcription levels of ORs can be significantly affected by prolonged exposure to high concentrations of their (best) ligands, we demonstrate limitations of a universal applicable DREAM method for deorphanization of different types of chemosensory receptors in insects.

## Materials and Methods

### Animals

All flies used in the experiments were WT *D. melanogaster* and belonged to the Canton-S strain (WTcs, stock #1), which was obtained from the Bloomington Drosophila Stock Center ^1^. The fly stock was maintained on an artificial diet at 25°C and 70% R.H. with a photoperiod of 12 h:12 h Light:Dark (Stökl et al., 2010). With the exceptions of the pheromone treatment and corresponding control groups, flies were collected 0–3 h after exclusion, pooled to groups of 60 and transferred to a fresh rearing vial. In case of exposure to the pheromone methyl laurate and for the corresponding controls, newly emerged flies were collected 5 days prior to the odorant exposure. On the day of the odorant treatment, the 5-days old flies were transferred to a fresh rearing vial for the exposure. Flies for all odorants were of mixed sex at a ratio of 1:1 and kept together during the length of the odorant exposure.

### Chemicals

Odorants used for all experiments were of highest purity commercially available and purchased from Sigma-Aldrich with the exception of methyl butyrate with was purchased from FLUKA. For the DREAM method, general odorants were diluted to 5% vol/vol in dimethyl sulfoxide (Sigma-Aldrich), while methanol (Roth) was used to dilute methyl laurate up to 5% vol/vol. In SSRs, odorant dilutions of 10^−4^ and 10^−1^ were used, dilutions were generated in hexane (Roth) for all general odorants and in methanol (Roth) for methyl laurate.

### Odorant Exposure and Tissue Collection

In order to test, whether the DREAM method is suitable for the deorphanization of broadly as well as narrowly tuned ORs and different chemosensory receptor types transcription changes of receptor genes were measured after flies were exposed to high odorant concentrations. Three hours after the beginning of the light phase the odorants or pure solvents were introduced into the rearing vials. To avoid interaction of the chemicals with the artificial diet, 30 μL of the odorants or solvents, respectively, were applied into the well of a detached 2.0 mL reaction tube lid. After an exposure time of 5 h, flies were transferred into new, empty vials, and cooled down for 5 min in a −80°C freezer. The flies were then maintained at −20°C until dissection. For each biological replicate, 50 manually removed fly heads (male–female ratio 1:1) were collected in 2.0-mL microcentrifuge tubes containing mixed zirconium oxide beats of 1.4 and 2.8 mm (CKmix-2 mL, Bertin Instruments) as well as 600 μL TRIzol^®^ (Sigma-Aldrich). For the sample collection of the pheromone treatments and corresponding controls, only the heads of male flies were used. During dissection, samples were stored on ice. After dissection samples were homogenized in a bead mill (TissueLyser LT, Qiagen) for 10 min at 50 Hz. Samples were centrifuged for 1 min at 13,000 *g* and stored at −80°C until RNA extraction.

### RNA Extraction and cDNA Synthesis

Total RNA for each replicate and treatment was extracted using an unbiased RNA isolation kit (Direct-zol^TM^ RNA MiniPrep, Zymo Research). The kit included a RNase-free DNase treatment to remove genomic DNA contamination from the samples. RNA concentration was measured with a *NanoDrop*^TM^ spectrophotometer (Thermo Fisher Scientific). First strand cDNA was generated from 1.0 μg of total RNA, using oligo-dT_20_ primers and superscript^TM^ III (Thermo Fisher Scientific). Subsequently, remaining RNA was digested via a RNase H treatment (Thermo Fisher Scientific).

### qPCR

Expression levels of target genes were analyzed by reverse transcription-mediated quantitative real-time PCR (qPCR). Following the guidelines proposed to guarantee reproducible and accurate measurements, qPCR reactions were run in a Stratagene Mx3005P qPCR system. Measurements were performed in 96-well plates using the Takyon^TM^ No Rox SYBR^®^ MasterMix dTTP blue (Eurogentec, Belgium) in a total reaction volume of 20 μL. Each reaction was run in triplicate with at least five independent biological replicates for controls and different treatments. Gene-specific primers for Cam, Orco, OR49b, OR67c, as well as OR82a were identical to those used in von der Weid et al. (2015). All other gene-specific primers were designed in Geneious (9.1.5). Primers are listed in **Supplementary Table S1**. The two-step thermal cycling protocol consisted of following steps: initial denaturation (95°C: 3 min), subsequent 40 cycles of denaturation (90°C: 10 s), annealing (60°C: 20 s), elongation (75°C: 30 s), and completed with a final cycle for post-amplification melting-curve analysis. The Cam and Orco genes were used as reference genes. For every primer pair used qPCR efficiency was determined by generating standard curves with mixed cDNA samples. Normalized expression and relative fold change were calculated based on a model by Vandesompele et al. (2002) for normalization against several reference genes when efficiencies of target and reference genes are not similar. Following equation from Vandesompele et al. (2002) was used for the calculations (E: primer efficiency, Cq: threshold cycle, Ref_*xy*_: reference gene, Tar: target gene):

$$\mathrm{ratio}=\frac{\sqrt[{n}]{{\left(1+E_{\mathrm{Ref}1}\right)}^{\mathrm{Cq}(\mathrm{Ref}1)}\times {\left(1+E_{\mathrm{Ref}2}\right)}^{\mathrm{Cq}(\mathrm{Ref}2)}\times \ldots }}{{\left(1+E_{\mathrm{Tar}}\right)}^{\mathrm{Cq}(\mathrm{Tar})}}$$

### Single Sensillum Recordings

In order to confirm the published ligand–receptor interactions, SSRs were performed with the same panel of odorants used in the DREAM experiments for all OSN types expressing the chemosensory receptors of interest. Flies of 2- to 7-day-old age were prepared for recordings as described by Clyne et al. (1997) and de Bruyne et al. (2001). With the help of a microscope (10× magnification, 0.30 numerical aperture [NA], Olympus BX51W1) and a micromanipulator (Märzhauser DC-3K) the reference electrode (tungsten wire) was manually inserted into one of the fixated fly’s compound eye. Next, changing the magnification to 50× (0.50 [NA]) and using a motorized, piezo-translator-equipped micromanipulator (Märzhauser DC-3K/PM-10), the recoding electrode (tungsten wire) was inserted into the center or shaft of a sensillum. Different OSN types localized inside the sensillum were identified using a set of known, well-established diagnostic odorants (Ebrahim et al., 2015). Spiking frequency of the OSNs expressing chemosensory receptors of interest was recorded for 10 s, starting 3 s before the stimulus (0.5 s stimulus duration), and lasting 7 s after the end of the stimulus. Neuronal signals were converted from a high input resistance to low-output resistance with a pre-amplification step (10×) using a headstage (Syntech Universal AC/DC probe). The pre-amplified signal was then converted (Syntech IDAC-4) and fed into a computer for visualization and analysis via Syntech Autospike v3.2. In order to discriminate between the neural activity of OSNs housed in the same sensillum, spikes were sorted by differences in their amplitude and assigned to distinct OSN types. Spikes with the largest amplitude were considered to belong to the OSN of type A, spikes of the second largest amplitude were assumed to originate from the B OSN and so forth. The amplitude-based spike sorting by Syntech Autospike v3.2 was manually adjusted when amplitudes of co-located OSNs changed after strong odorant stimulation. In cases where amplitudes between OSNs housed in the same sensillum were not distinguishable due to extensive neural activity, the final spike frequency represents the total response of a sensillum. The electrophysiological data was analyzed by subtracting responses to the control solvents from each observed odorant response stimulus (decrease or increase in firing frequency) for each tested chemosensory receptor.

### GC–MS Headspace Analysis

As the exposure to the different odorants during the DREAM experiments lasted for several hours, we confirmed by GC–MS analyses that all test compounds were chemically stable and present in high amounts during the whole exposure period. Solvents or diluted odorants were placed into fly vials with artificial diet, simulating the experimental conditions in absence of the actual flies while additionally analyzing the effect of the presence of fly food on the odorant profile. The headspace in the experimental setup was collected for 5 min with a SPME microfiber (StableFlex^TM^, DVB/CARBOXEN-PDMS, Supelco) after 45 min and 4 h of introducing the solvent or odorant into the system. Headspace samples were manually injected into a GC–MS device (Agilent technologies GC 6896N interfaced with an Agilent technologies 5975B inert XL MSD unit) with an installed HP-5MS UI column (19091S-413U, Agilent technologies). For sample analysis, the temperature of the gas chromatograph oven was held at 40°C for 2 min and then gradually increased by 20°C min^−1^ up to 260°C. Electron impact (EI) was measured at 70 eV and 300 μA in scan mode ranging from 33 to 350 m/z. The temperature of the transfer line was held at 280°C, and the ion source was maintained at 230°C. GC–MS profiles of all headspace samples were interpreted by comparison to a standard library (NIST Mass spectrum library) using MSD ChemStation (F.01.02.2357, Agilent).

### Analysis of Transcription Levels

**FIGURE 1:**
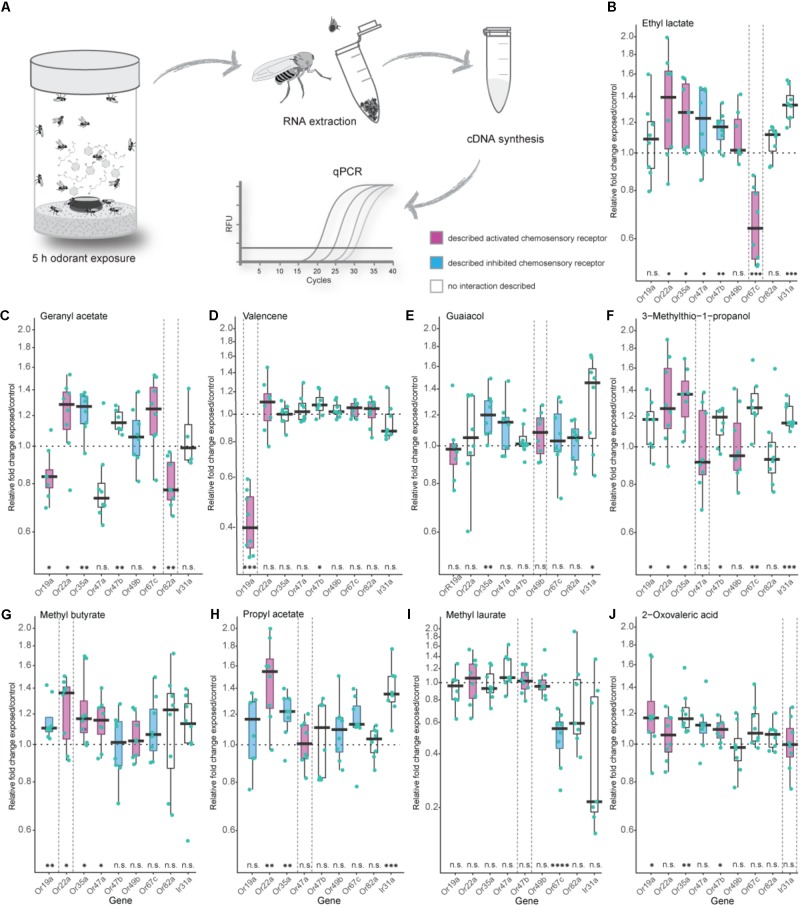
Effects of exposure to high odorant concentration on transcription levels of selected chemosensory receptors. **(A)** Experimental procedure from odorant treatment to a final analysis of gene transcript-levels via quantitative real-time PCR (qPCR). **(B–J)** Evaluation of chemosensory mRNA levels after 5 h exposure to 5 % v/v of depicted odorants using qPCR. Each data point represents a biological replicate with a pool of RNA from 50 fly heads of mixed sex (ratio 1:1), except in **(I)** where heads from males only were used. For every odorant treatment the number of biological replicates was eight with the exception of the Ir31a gene in the geranyl acetate treatment series **(C)** where *n* = 4. Best ligand–receptor pairs are highlighted between dotted vertical lines. Excitatory and inhibitory odorant interactions (doOR database and measured in this study) are indicated in magenta (excitatory) and cyan (inhibitory). Boxplots represent the median (bold horizontal lines) with the interquartile range (whiskers). Results from a One sample *t*-test against 1 are shown on the *x*-axis. Asterisks indicate significant differences (^∗^*P* < 0.05; ^∗∗^*P* < 0.01; ^∗∗∗^*P* < 0.001; ^∗∗∗∗^*P* < 0.0001).

For the analysis of possible regulations in mRNA levels of chemosensory receptors upon prolonged exposure to 5% v/v of odorants, we calculated the significance of relative fold changes in gene mRNA levels that were different from 1 based on One sample *t*-tests (**Figure 1**, *x*-axis). Additionally, for comparison to the original study, we defined an unresponsive zone using the data points of published unresponsive chemosensory receptors to apply a Gaussian distribution to the data set [**Supplementary Figure S1**, gray area (von der Weid et al., 2015)]. Following instructions from von der Weid et al. (2015), the unresponsive zone was defined within 1.4 σ above and below the mean based on the results from the Gaussian fit for all the different odorant treatment series. All data points inside the unresponsive zone were considered as treatment independent variations in mRNA levels and thus not relevant while data points outside were regarded as alterations in mRNA levels caused by the odorant exposure and therefore relevant (**Supplementary Figures S1**, **S2**).

Analysis of our gene mRNA levels in regards to significant fold changes different from 1 as well the definition of an “unresponsive zone” led to similar conclusions. We, hence, focused on analyzing our results looking for fold changes different from 1.

## Results

### Specificity of Chemosensory Receptor mRNA Level Alterations After Odorant Exposure

**FIGURE 2:**
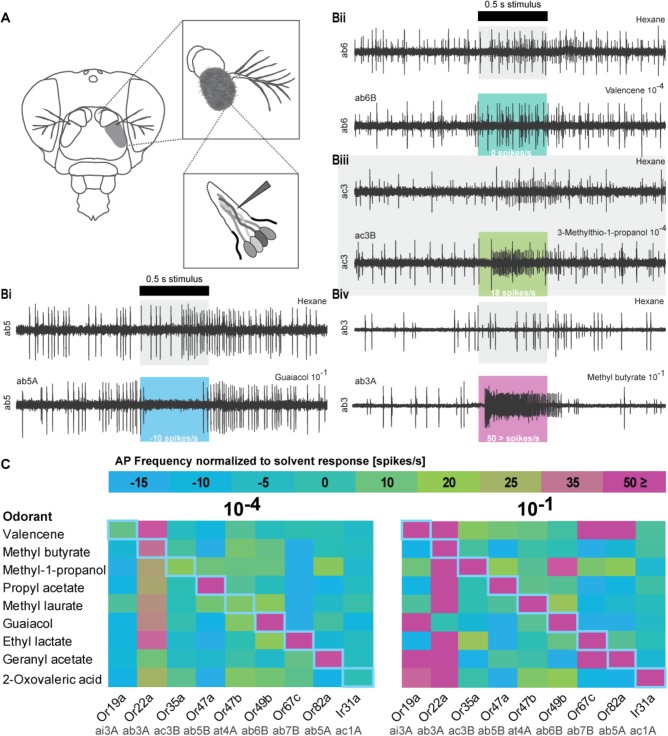
Olfactory sensory neuron (OSN) responses after stimulation with odorants used in the DREAM technique. **(A)** Schematic drawing of Single Sensillum Recording (SSR) procedure. (Bi-Biv) Representative SSR traces of sensilla activity upon presentation of the respective solvents, an inhibitory interaction (guaiacol 10^−1^, ab5A), no interaction (valencene 10^−4^, ab6B), an excitatory interaction (3-methylthio-1-propanol 10^−4^, ac3B), and a highly excitatory interaction (methyl butyrate 10^−1^, ab3A). The black bar marks stimulus delivery and duration (0.5 s). Colored boxes correspond to heat map in **(C)**. The OSN response after subtraction of possible solvent responses is stated on the bottom of each colored box. **(C)** Color-coded average responses i.e., frequency of action potentials (AP) measured in SSRs to stimulation with odorants at a 10^−4^ and a 10^−1^ dilution (*n* ≥ 4). Changes in spontaneous chemosensory receptor activity induced by used solvents have been subtracted from the recorded firing frequency. Numerical values can be found in **Supplementary Table S2**. Blue diagonal line represents expected, ideal results based on original publication (von der Weid et al., 2015).

In *Drosophila melanogaster*, the majority of ORs and IRs have been functionally analyzed, and their ligand spectra have been characterized using electrophysiological approaches like SSR in wildtype flies as well as in mutant flies with different “empty neuron” or “decoder” systems. The doOR (**d**atabase **o**f **o**dorant **r**esponses) online platform provides an extensive database for known ligand–chemosensory receptor pairs^2^. First, we established the DREAM technique in our laboratory by reproducing the results from the original study [**Figures 1B,C** (von der Weid et al., 2015)]. When we exposed 0 to 3 h-old flies to the described best ligand for Or67c and Or82a, that is, ethyl lactate and geranyl acetate, respectively, we observed a significant reduction in the mRNA levels of these genes at the end of the treatment. Long-time exposure to 5% v/v ethyl lactate resulted in a downregulated transcription only of the target receptor Or67c. However, exposure to geranyl acetate did not only downregulate the transcription of Or82a as expected, but also interestingly of Or47a (**Figure 1C**), which is expressed in an OSN that is co-localized in the same sensillum as the OSN expressing Or82a. Moreover, we observed a significant decrease in gene transcription of Or19a after geranyl acetate treatment (**Figure 1C**). While SSR measurements revealed that OSNs expressing Or19a indeed become activated by high amounts of geranyl acetate, Or47a does not seem to have any interaction with this odorant (**Figure 2C**).

Next, we tested the specificity of the DREAM technique with valencene, an odorant previously known to only activate two ORs of the OR repertoire of *Drosophila melanogaster*, that is, Or19a and Or71a (Dweck et al., 2013; Ronderos et al., 2014). Of these two receptors, Or19a shows a substantial activation by valencene, accompanied by a very strong increase in the firing rate (spikes/s) after valencene stimulation of the ai2 sensillum (Dweck et al., 2013). Thus, we chose Or19a as the next target OR to test the specificity of the DREAM technique. Exposure of flies to valencene decreased Or19a mRNA levels substantially and exclusively (**Figure 1D**). None of the other tested ORs showed any reduction in transcription.

We further analyzed the specificity of the DREAM technique by testing a ligand–receptor combination [Or49b and guaiacol (Dweck et al., 2015a)] in which the OR has been identified to possess a narrowly tuned ligand spectrum (de Bruyne et al., 2001; Hallem and Carlson, 2006; Marshall et al., 2010). Surprisingly, after the odorant treatment Or49b mRNA levels did neither show a downregulation nor a significant upregulation (**Figure 1E**). While exposure to guaiacol did not have a measurable influence on the expression levels of the Or49b gene, stimulation with this odorant did lead to an increase in the firing activity of ab6B OSNs in SSRs (**Figure 2C**), confirming the published ligand–receptor interaction of guaiacol and Or49b. We therefore asked whether a lack in downregulation of the receptor’s transcription might be due to degradation of guaiacol during the duration of the odorant treatment. However, an analysis of the headspace in the experimental setup confirmed that guaiacol did not break down into other compounds and was present at an abundance comparable to those of ethyl lactate, geranyl acetate, and valencene (**Supplementary Figures S3A–D**).

Subsequently, we wanted to ascertain the effects of prolonged exposure to broadly activating odorants on the transcription of OR genes. High concentrations of odorants, comparable to rates used in the DREAM technique, have been shown to elicit, possibly unspecific, increases in the firing frequency of different broadly tuned OSNs, while lower concentrations of the same compound do not activate these OSNs to a significant degree (Hallem and Carlson, 2006; Kreher et al., 2009). Thus, we were interested in learning if broadly activating odorants used with the DREAM technique cause unspecific up- or downregulation in OR mRNA levels, particularly of those receptors being characterized as broadly tuned. Exposure to neither 3-methylthio-1-propanol, methyl butyrate, nor propyl acetate did coincide with a significant downregulation of any of the tested chemosensory receptor genes (**Figures 1F–H**). Instead, we observed significant increases in transcript levels of those chemosensory receptors, which are described to be either activated or inhibited by the tested odorants (**Figures 1F**–Or22a, Or35a; G–Or19a, Or22a, Or35a, Or47a; H–Or22a, Or35a). Interestingly, odorant treatment with 3-methylthio-1-propanol and propyl acetate did also lead to an upregulation in expression levels of chemosensory receptors electrophysiologically characterized as being unresponsive to these compounds (**Figures 1F**: Or19a, Or47b, Or67c, Ir31a; H: Ir31a).

So far, we had examined the effects of exposure to general, fruit and host odorants on the transcription levels of general ORs, but we were also interested in looking at a possible correlation between exposure to pheromones and changes in regulation patterns of the corresponding PRs. In *D. melanogaster*, OSNs expressing PRs have been shown to exhibit an age-dependent sensitization toward their ligands reaching a maximum after 7 days (Lin et al., 2016). Thus, instead of the previously used 0 to 3 h-old flies we used 5-days-old flies in our pheromone treatment series, in which we exposed the flies to 5% v/v of methyl laurate, a pheromone activating Or47b (Dweck et al., 2015b; Lin et al., 2016). When flies were exposed to 5% v/v of methyl laurate expression levels of the monitored chemosensory receptors, including Or47b, remained unchanged with the exception of Or67c mRNA levels, which were downregulated (**Figure 1I**). Again a screen for methyl laurate in the headspace of the experimental setup validated the presence of the odorant from the beginning to the end of the experiment at an abundance similar to those causing changes in the transcription levels of ORs in prior treatment series (**Supplementary Figure S3G**).

Finally, we were curious to learn if the DREAM technique could be utilized to deorphanize members belonging to the chemosensory receptor type family of IRs. The application of the DREAM technique for odorants activating IRs is limited by the chemical properties of the different odorants. At odorant concentrations used in the experimental setup of the DREAM method, the compounds can develop a deleterious influence on the health of the treated flies, possibly leading to unspecific changes in the expression levels of a plethora of genes, including those of chemosensory genes, and/or the death of the tested flies. Hence, we focused on 2-oxo-valeric acid and Ir31a, an IR-ligand combination in which the odorant has no critical impact on the health of flies in the treatment group (**Supplementary Table S4**). Exposure to 2-oxo-valeric acid did not lead to changes in the transcription of the IR31a gene or of the Or22a gene both being chemosensory receptors shown to be activated by stimulation with this odorant [**Figures 1J**, **2C** (Silbering et al., 2011)]. However, we did observe an upregulation in the transcription level of Or19a as an OR being gated by 2-oxo-valeric acid but also an increase in transcription of Or35a and Or47b, both ORs being unresponsive to stimulation with this odorant in SSR (**Figure 2C**).

**FIGURE 3:**
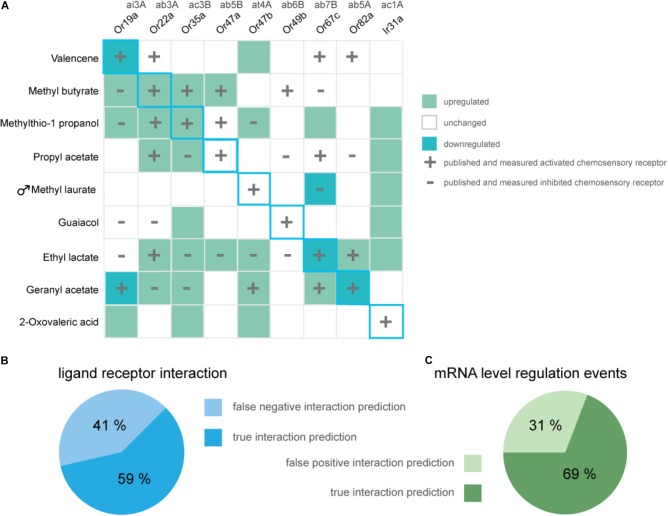
Overview of alterations in chemosensory receptor gene transcript levels after odorant exposure. **(A)** Shown are significant increases (dark green) and decreases (light green) in transcription of chemosensory receptors *in Drosophila melanogaster* flies which were exposed to 5% v/v of displayed odorants for 5 h (One sample *t*-test; *n* = 8, exception Ir31a- geranyl acetate, *n* = 4). Unchanged expression levels are depicted in white. Data for previously characterized interactions of an odorant and chemosensory receptor are indicated with a plus (excitatory interaction) or a minus (inhibitory interaction), respectively. Blue diagonal line represents expected, ideal results based on original publication (von der Weid et al., 2015). **(B)** The pie chart as a whole represents all published and reproduced ligand–receptor interactions for odorants used in the DREAM treatments [plus and minus symbols in **(A)**], visualized are the percentages of overlaps between alterations in mRNA levels and Single Sensillum Recording data (“true interaction prediction”) as well as unchanged receptor mRNA levels despite electrophysiologically proven ligand–receptor interactions (“false negative interaction prediction”). **(C)** Visualized are the percentages of all measured changes in receptor mRNA levels which show an overlap with published data for ligand-receptor interactions (“true interaction prediction”) or which have occurred without electrophysiological proof for a ligand-receptor interaction (“false positive interaction prediction”).

In summary, from eight tested general odorants, in three cases prolonged exposure to 5% v/v of the described (best) ligand successfully resulted in a decrease in transcription of the corresponding chemosensory receptor (**Figure 3A**, light green squares in diagonal center line). Interestingly, independent of an excitatory or inhibitory ligand–receptor interaction, we did find gene regulation, mostly increases in transcription (**Figure 3A**, dark-green squares) of known interacting chemosensory receptors in all odorant treatment series (**Figure 3A**, overlap plus and minus symbol with colored squares). However, proven sensitivity to a certain odorant was no predictor for an alteration in mRNA levels of a chemosensory receptor analyzed with the DREAM technique (**Figure 3A**, plus or minus symbol no colored square). Of all described and measured known ligand–receptor interactions 59% of interactions were correctly predicted with the DREAM technique while 41% of interactions were falsely predicted to be negative (“false negative,” **Figure 3B**). In some cases, exposure to odorants lead to unspecific changes (“false positive,” **Figure 3C**) in transcription of chemosensory receptors prior being identified as unresponsive to those compounds (**Figure 3A**, colored square no plus or minus symbol). Furthermore, application of the DREAM technique to the previously untested chemosensory receptor types (i.e., PRs and IRs) did not result in changes in expression levels of described best ligand–receptor pairs, suggesting the DREAM technique is not applicable to these receptor types for novel deorphanization.

### Validation of Ligand–Receptor Pairings With Single Sensillum Recordings

When we found neither up- nor downregulation for some of the described responsive chemosensory receptors after prolonged odorant exposure in the DREAM method, we next performed SSR to confirm the sensitivity of our chemosensory receptors set to all tested odorants (**Figure 2**). Moreover, we wanted to ascertain if we could explain the detection of upregulation events for chemosensory receptors which have been previously described as being unresponsive to the corresponding odorant, with unspecific interactions or artifacts of the high concentrations used in the DREAM technique. We screened the receptor set in SSR with an ecologically relevant odorant dilution of 10^−4^ and an odorant dilution of 10^−1^ (the latter of which was similar to amounts applied within the experimental setup of the DREAM technique) (**Figure 2C**). Recorded spiking frequencies were assigned to the individual OSNs housed in the corresponding sensillum according to differences in spike amplitudes (for details see section “Materials and Methods”). All odorants used in the treatment groups elicited a substantial increase (≥50 spikes/s) in the frequency of OSN firing for the corresponding (best) ligand–receptor combination (**Figure 2C**: 10^−1^, magenta rectangles). Furthermore, we were not able to attribute all alterations in the transcription of ORs described as unresponsive to unspecific interactions at high odorant concentrations based on our SSR data. We could not elicit changes in the receptor’s firing frequency when stimulated with the corresponding odorant at a dilution of 10^−1^ (**Figure 1F**: Or19a, Or67c; C: Or47a, Or47b; J: Or35a). However, in two cases, we measured alterations in the expression levels of ORs that were previously characterized as being unresponsive to the tested odorant and observed discrepancies between published ligand spectra and response profiles based on our recordings at a dilution of 10^−1^ (**Figure 1C**: OR19a; J: Or47b). Finally, comparing the data generated from SSR with the expression levels of Ir31 after exposure to our nine tested odorants, we found that in all four cases of alterations in the IR gene’s transcription (**Figures 1A,E,F,H**), stimulation with those odorants did not change the spiking frequency of the OSN in SSR.

### Correlation of Alterations in Receptor Transcript-Levels to Either Excitatory or Inhibitory OSN Responses Upon Odorant Stimulation

After completion of our SSR screens, we were curious to learn if there was a correlation between receptor up- and downregulation following an excitatory or following an inhibitory interaction of the odorant used in the DREAM setup with the chemosensory receptor. In other words, does an excitatory ligand–receptor interaction lead to a decrease in mRNA levels of the corresponding receptor and an inhibitory interaction to an increase in transcription? We plotted the odorant treatment induced change in gene transcription against the increase or decrease in spiking frequency observed in SSR (**Supplementary Table S2**). Here, we found significant correlations between these two traits for all genes in which exposure to its best ligand did lead to a decrease in expression levels at odorant dilutions of 10^−1^ (Or19a, Or67c, and Or82a; **Supplementary Figure S4** and **Supplementary Figure S3**). However, these correlations depended solely on the data point for the described best ligand interaction. If those data points were excluded from the data pool, the two traits were no longer correlated.

## Discussion

The characterization of ligand spectra of chemosensory receptors in *Drosophila melanogaster* has led to a comprehensive database of chemo-signals causing receptor activation or inhibition. Data collection of the ligand spectra for the chemosensory receptor has been a huge undertaking and took tremendous efforts from various laboratories over several decades (Münch and Galizia, 2016). Furthermore, functional characterization of chemosensory receptors has only been possible due to the exceptional availability of numerous genetic tools, like ectopic receptor expression with the Gal4-UAS system (Brand and Perrimon, 1993), and not least due to the ease of accessibility to chemosensory OSNs for electrophysiological recordings. In other non-model insect species, scientists are still struggling to identify ligand–chemosensory receptor combinations as most genetic tools are not yet available. However, a high-throughput characterization of ligand–receptor interactions would highly facilitate the identification of active odorants and their corresponding neuronal circuits involved in mediating ecologically relevant behaviors such as host or mate choice. The recently established DREAM technique (von der Weid et al., 2015) has the potential to be such a tool.

Using published ligand–receptor pairs in *D. melanogaster*, we tested whether DREAM can be used as a reliable tool for the prediction of ligand–receptor interactions of six narrowly and broadly tuned general ORs, as well as one pheromone OR and one olfactory IR. In an ideal scenario, following the observation from the original study (von der Weid et al., 2015), we would have expected to find a downregulation in transcript levels for all eight of our ligand–OR pairs (**Figure 3**, diagonal blue outline). Moreover, if the decrease in expression levels was a general indicator for an excitatory interaction of odorant and receptor, we expected to measure a downregulation in mRNA levels not only for the interaction of a receptor and its best ligand but for all chemosensory receptors being activated by a corresponding compound (**Figure 3**, plus symbols).

We only found a decrease in gene transcript levels for three out of eight (best) ligand–OR combinations (**Figure 3A**, light green squares with blue outline). At the same time, we measured an increase in expression levels in two of the eight (best) excitatory ligand–OR pairs (**Figure 3A**; methyl butyrate-Or22a, methylthio-1-propanol-Or35a, dark-green squares with blue outline), while the expression levels of the remaining chemosensory receptors remained unchanged after odorant exposure. Taken together, we observed alterations in mRNA levels in five out of eight tested (best) ligand–OR pairings.

Following the conclusions of the original study (von der Weid et al., 2015), we hypothesized that the direction of changes in receptor mRNA levels (up- or downregulation) upon odorant exposure might be correlated to the mode of ligand–receptor interaction (inhibition vs. excitation). A current hypothesis is an adaptive modulating response of the OSN to possible excitatory overstimulation over an extended period of time, which would render the neuron less sensitive to lower odorant concentrations (von der Weid et al., 2015). This was, however, not observed in preliminary SSR experiments, where after 5–6 h of odorant exposure, the measured downregulation in OR gene transcript levels did not translate into changes of the corresponding OR’s dose-response curve to the tested odorant (data not shown). The exact duration of conversion of “transcript to protein” is not known for each OR, and it is possible that the protein synthesis occurred in a different time window than what was monitored. Nevertheless, in several cases we also observed increases in the amount of OR mRNA levels after exposure to excitatory odorants; therefore, a simple adaptive, desensitizing response due to overstimulation seems unlikely (e.g., **Figure 3**, methyl butyrate: Or22a, methylthio-1-propanol: Or35a). We conclude that excitation of an OSN with its best ligand does not necessarily result in downregulation of gene transcription of the neuron’s corresponding chemosensory receptor. For some ligand–receptor pairs, we found upregulation in gene transcription independent of receptor excitation or inhibition. We thus infer that alterations in chemosensory receptor expression levels following the DREAM technique are not indicative of the nature of the ligand–receptor interaction.

Since the modulation mechanisms induced by prolonged odorant exposure are not known, a correlation of the direction of alterations in receptor mRNA levels to other factors than the mode of ligand–receptor interaction are worth to consider: for instance a correlation to the ligand–receptor binding properties of odorant to the OR followed by possible induced conformational changes in the receptor, leading to differences in the receptor’s properties. These induced changes in the receptor’s characteristics could then define whether the odorant treatment of the DREAM method leads to an increase or decrease in mRNA levels of the corresponding receptor.

When we compared all characteristics and properties of those ORs where we observed gene transcription alterations upon odorant treatment, we were not able to find a common thread that would connect a successful application of the DREAM method, such as sensillum type, receptor specificity or chemical properties of the used ligands.

For all ligand–receptor pairings that did not show alterations in gene transcript levels (either up- or downregulation), we were still able to confirm active ligand-receptor interactions in SSR measurements. Additionally, we analyzed odorant stability and concentration during the long-term exposure experiments using SPME and we were able to demonstrate odorant integrity as well as presence at high concentrations until the end of the treatment (4 h exposure duration; **Supplementary Figure S3**). Therefore, we can exclude that the lack of alterations in gene transcription was due to inadequate ligand–receptor pairs or deficient odorant stimulation, as each odorant stimulated the receptor of interest and persisted without degradation at high concentration throughout the exposure duration of the DREAM method.

While we were not able to reproduce the trend of correlating an excitatory interaction to a reduction in chemosensory receptor mRNA levels for all ligand–receptor pairs and likewise inhibitory interactions to increases in receptor gene transcription, in 69% of observed regulatory events (**Figure 3C**), we found an overlap between expression alterations and electrophysiologically measurable ligand–receptor interaction.

A possible explanation for “false positives,” that is, alterations in mRNA levels although the ligand did not interact with the receptor in SSRs (**Figure 3C** “false positives,” e.g., **Figure 1D**: Or47b), could be due to the fact that odorant concentrations in neither our, nor the electrophysiological recordings from available datasets, were as high as those used in the DREAM technique. High concentration stimuli are not occurring in nature and thus outside the typical bounds of receptor function. A critical influence of odorant concentrations on the extent of changes in receptor mRNA levels was already noticed by the authors of the original study (von der Weid et al., 2015). It is thus possible that at concentrations present in the DREAM experimental setup, unspecific odorant and receptor interactions would occur, causing the observed “false positive” alterations in chemosensory receptor mRNA levels.

In cases in which we were not able to find alterations in gene transcript-levels upon odorant exposure despite having evidence for a ligand–receptor interaction (“false negative” predictions, i.e., no alterations in gene mRNA levels although the ligand did interact with the receptor in SSRs), the exposure duration of 5 h may have been too short to induce changes in gene transcription. When we tested this assumption and increased the exposure time to 10 h for guaiacol and its corresponding receptor Or49b, we did indeed find a tendency toward downregulation of transcription (**Supplementary Figure S5**). Moreover, data from applications of the DREAM method in mice shows that the maximum impact of odorant exposure on the mRNA levels of the corresponding receptor occurs at different hours after the treatment has started, varying between tested ORs (von der Weid et al., 2015). Hence, exposure duration during the DREAM technique appears to be a critical factor that might have to be modified and adjusted for every ligand–receptor interaction, making the technique less applicable.

An additional factor that might hamper the applicability of the DREAM technique for the deorphanization of some olfactory receptors could be differences in transcriptional variability between genes in the olfactory system of *D. melanogaster*. Some ORs might underlie a strict expression and transcript-level control, while other ORs might be less tightly regulated. Transcript-levels of ORs could be regulated differentially between individual OR genes or OR gene groups via distinct post-transcriptional mRNA features, regulating translational repression and mRNA stability (Shum et al., 2015). The relatively small changes in gene mRNA levels following the DREAM treatment would be less prominent on the background of an already high transcriptional variability, making these alterations harder to detect.

There is thus room for customizing the parameters of the DREAM technique to expand its applicability to a broader set of ORs, perhaps even other chemosensory receptor classes like IRs. A starting point for modifications to the parameters of the DREAM method could be the choice of reference genes since we found at least effects of exposure to 3-methylthio-1-propanol on the expression of the reference gene ORCO (**Supplementary Table S5**). Adjusting the DREAM method in regards to odorant concentration or exposure duration in order to find alterations in the corresponding receptor’s gene transcription in *D. melanogaster* is only possible due to the availability of extensive databases for ligand–receptors pairs, as it is known exactly which receptors should be affected by which ligand. In most other insect species such databases are of course not available. In the vinegar fly, the identification of new ligand–receptor combinations using the DREAM technique is also hindered by the fact that, according to our findings, there are false positive or non-specific regulatory events that can occur and some ORs seem to be unresponsive to the odorant treatment in regard to differences in expression. In mouse and rat the amount of “false positive” as well as “false negative” ligand–receptor interactions observed after odorant exposure was negligible (0%, von der Weid et al., 2015), and ORs proven to be activated by a ligand *in vivo* also consistently demonstrated alterations in transcript levels (Jiang et al., 2015; von der Weid et al., 2015; Ibarra-Soria et al., 2017). Hence, the DREAM technique seems to be well suited for the deorphanization of ORs in mammals, but less so in insects. When applied to an insect system for identification of possible ligand interaction partners, the relatively high amount of false positive predictions (31%) produced by the DREAM technique is less serious since these predictions when tested in heterologous expression systems or *in vivo* are easy to be characterized as false. However, the even higher amount of false negative predictions (41%) has a more severe impact on the applicability of the technique. The inability of detecting all interaction partners would lead to wrong and/or limited conclusions about ligand–receptor pairings and could prevent the elucidation of important ligand interaction partners.

## Conclusion

We confirmed the findings from the original study on *D. melanogaster* regarding the down-regulation of Or67c and Or82a upon exposure to their corresponding agonists, ethyl lactate and geranyl acetate, respectively. However, based on our additional results from a broader array of ORs, it seems highly unlikely that the application of the same experimental conditions during the DREAM treatment will work for the deorphanization of a large set of ORs, neither in *D. melanogaster*, nor in other insect species where novel deorphanization is necessary. Further analyses of cases where DREAM does appear successful (such as Or19a-valencene and Or67c-ethyl lactate), may provide more rationale as to where and when this technique can be utilized or as to which parameters have to be modified for a reliable ligand–receptor interaction prediction. Consequently, with the current flaws in the applicability of the DREAM technique there is still no way around time-consuming olfactory receptor deorphanization via the well-established “empty neuron” or “decoder systems” and functional characterization in heterologous expression systems.

## Author Contributions

BH, MK, and SK conceptualized the study. MK, EG, SK, and LC planned the qPCR experiments. SK carried out all applications of the DREAM technique, the steps leading up to the qPCR experiments, and the qPCR runs themselves. LC and SK analyzed the qPCR data. IK, SK, and MAK performed electrophysiological recordings. SK carried out GC–MS data collection and analysis and performed computational analysis. SK and MK wrote the first draft of the manuscript and all authors reviewed and edited the manuscript.

## Conflict of Interest Statement

The authors declare that the research was conducted in the absence of any commercial or financial relationships that could be construed as a potential conflict of interest.

## Footnotes

1 https://bdsc.indiana.edu

2 neuro.uni-konstanz.de/DoOR/default.html

## Supplementary Material

FIGURE S1 related to Figure 1 Comparison of chemosensory receptor mRNA levels after long-time odorant exposure **(A–J).** Analysis of receptor transcript-levels after DREAM treatment implementing two different approaches for the statistical interpretation of observed fold changes. Boxplots show the median (bold horizontal lines) with the interquartile range (whiskers). On the *x*-axis results from a One sample *t*-test against 1 are shown. Asterisks indicate significant differences (^∗^*P* < 0.05; ^∗∗^*P* < 0.01; ^∗∗∗^*P* < 0.001; ^∗∗∗∗^*P* < 0.0001). The gray area represents the “unresponsive zone,” which is calculated based on a Gaussian distribution for all data points from described unresponsive chemosensory receptors to stimulation with the applied odorant (for more details see Materials and Methods). Each biological replicate, which includes a pool of RNA from 50 fly heads of mixed sex (ratio 1:1) or heads from males only **(F)**, is represented by one data point. For every odorant treatment the number of biological replicates was eight with the exception of the IR31a gene in the geranyl acetate treatment series **(B)** where *n* = 4. Highlighted between the two dotted lines are the best ligand–receptor pairs for the corresponding odorant treatment. Excitatory and inhibitory odorant interactions (doOR database and measured in this study) are indicated in magenta (excitatory) and cyan (inhibitory).

FIGURE S2 related to Figure 3 Overview significant fold changes in chemosensory receptor mRNA levels when unresponsive zone is applied. Alterations induced by exposure to 5% v/v of displayed odorants in chemoreceptor gene transcription, which remain significant after application of an “unresponsive zone” (for more details see methods) are indicated in dark green (upregulation) and light green (downregulation). Unchanged expression levels are depicted in white. Data for previously characterized interactions of an odorant and chemosensory receptor are indicated with a plus (excitatory interaction) or a minus (inhibitory interaction), respectively. Blue diagonal line represents expected, ideal results based on original publication (von der Weid et al., 2015).

FIGURE S3 Odorant integrity remains unchanged and odorant abundance is stable during long-time exposure of DREAM treatment **(A–I)** GC-MS profiles of the headspace in fly rearing vials without fly food and flies after 45 min (white background) and 4 h (light green background). In each panel, the odorant used in the DREAM treatment series is highlighted together with its solvent dimethyl sulfoxide (DMSO), with the exception of methyl laurate in which case the solvent methanol was already completely evaporated before the first headspace was collected **(G)**.

FIGURE S4 Alterations in chemosensory receptor mRNA levels upon odorant treatment cannot generally be used to predict the nature of ligand-receptor interactions. **(A–J)** Correlation of up- and downregulation events in mRNA levels of Or19a, Or22a, Or49b, Or67c, and Or82a against olfactory sensory neuron spiking frequency upon odorant exposure and odorant stimulation at a dilution of 10^−4^ or 10^−1^, respectively. Data points represent the individual odorants tested in the DREAM experiments. In each panel, the gray area indicates a confidence interval of 95%.

FIGURE S5 A 10 h odorant exposure to guaiacol indicates a trend to mRNA level downregulation of the best ligand–receptor pairing which was previously unresponsive after an exposure duration of 5 h. Evaluation of chemosensory receptor mRNA levels after exposure to 5% v/v guaiacol for 10 h with qPCR. Each data point represents one biological replicate and consists of a pool of RNA from 50 *Drosophila melanogaster* heads with a mixed sex ratio of 1:1. The total amount of biological replicates equaled six. On the *x*-axis results from a One sample *t*-test against 1 are shown. Asterisks indicate significant differences (n.s. *P* > 0.05; ^∗^*P* < 0.05). Highlighted between the two-dotted lines is the best described chemosensory receptor for guaiacol. A cyan box indicates a described and measured inhibitory ligand–receptor interaction while a magenta box refers to a described and measured excitatory ligand-receptor interaction.

TABLE S1 Primers used in qPCR experiments. Asterisks indicate primers identical to those from von der Weid et al. (2015).

TABLE S2 related to Figure 2 Numerical values of Single Sensillum Recording measurements in spikes/s for odorants used in the DREAM treatment at a dilution of 10^−4^ and 10^−1^.

TABLE S3 Results of a correlation analysis between up- and downregulation in receptor mRNA levels and spiking frequency of olfactory sensory neurons expressing indicated receptor upon stimulation with odorants tested in the DREAM experiments.

TABLE S4 Survival rate of 50 *D. melanogaster* flies exposed to dimethyl sulfoxide only (DMSO; control) or 5% v/v of oxovaleric acid in DMSO after 24 h in the DREAM experimental setup.

TABLE S5 Differences between Cq (threshold cycle) of control and treatment samples for the two reference genes CAM and ORCO.
