# Supplementary figures and images for: Evaluation of the DREAM Technique for a High-Throughput Deorphanization of Chemosensory Receptors in Drosophila
Source: Front Mol Neurosci. 2018 Oct 9;11:366. doi: 10.3389/fnmol.2018.00366 (PMC6189519; doi:10.3389/fnmol.2018.00366)

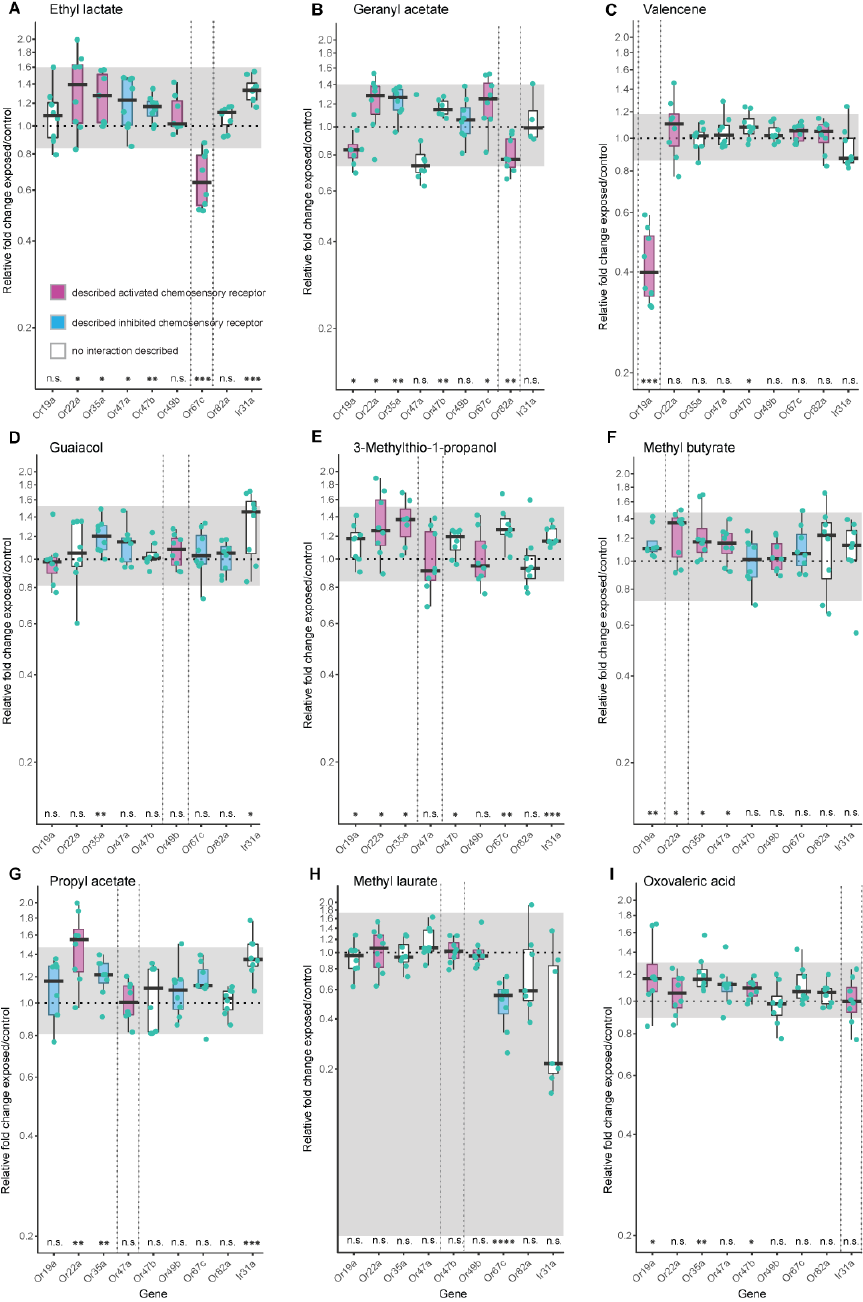

Supplement: FIGURE S1 related to Figure 1 — Comparison of chemosensory receptor mRNA levels after long-time odorant exposure (A–J). Analysis of receptor transcript-levels after DREAM treatment implementing two different approaches for the statistical interpretation of observed fold changes. Boxplots show the median (bold horizontal lines) with the interquartile range (whiskers). On the x-axis results from a One sample t-test against 1 are shown. Asterisks indicate significant differences (∗P < 0.05; ∗∗P < 0.01; ∗∗∗P < 0.001; ∗∗∗∗P < 0.0001). The gray area represents the “unresponsive zone,” which is calculated based on a Gaussian distribution for all data points from described unresponsive chemosensory receptors to stimulation with the applied odorant (for more details see Materials and Methods). Each biological replicate, which includes a pool of RNA from 50 fly heads of mixed sex (ratio 1:1) or heads from males only (F), is represented by one data point. For every odorant treatment the number of biological replicates was eight with the exception of the IR31a gene in the geranyl acetate treatment series (B) where n = 4. Highlighted between the two dotted lines are the best ligand–receptor pairs for the corresponding odorant treatment. Excitatory and inhibitory odorant interactions (doOR database and measured in this study) are indicated in magenta (excitatory) and cyan (inhibitory). [file Image_1.tif]

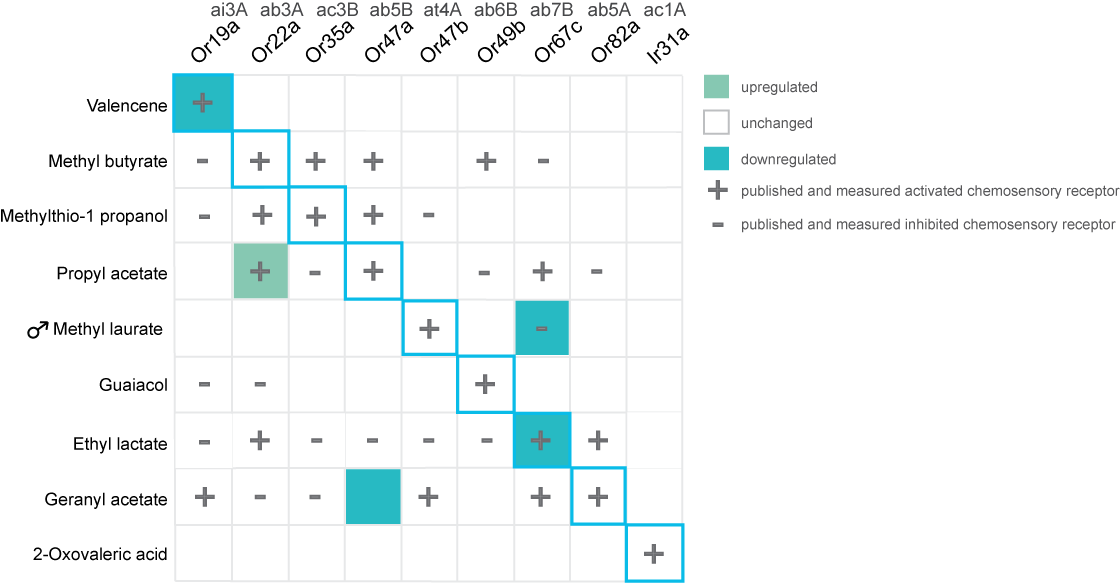

Supplement: FIGURE S2 related to Figure 3 — Overview significant fold changes in chemosensory receptor mRNA levels when unresponsive zone is applied. Alterations induced by exposure to 5% v/v of displayed odorants in chemoreceptor gene transcription, which remain significant after application of an “unresponsive zone” (for more details see methods) are indicated in dark green (upregulation) and light green (downregulation). Unchanged expression levels are depicted in white. Data for previously characterized interactions of an odorant and chemosensory receptor are indicated with a plus (excitatory interaction) or a minus (inhibitory interaction), respectively. Blue diagonal line represents expected, ideal results based on original publication (von der Weid et al., 2015). [file Image_2.TIF]

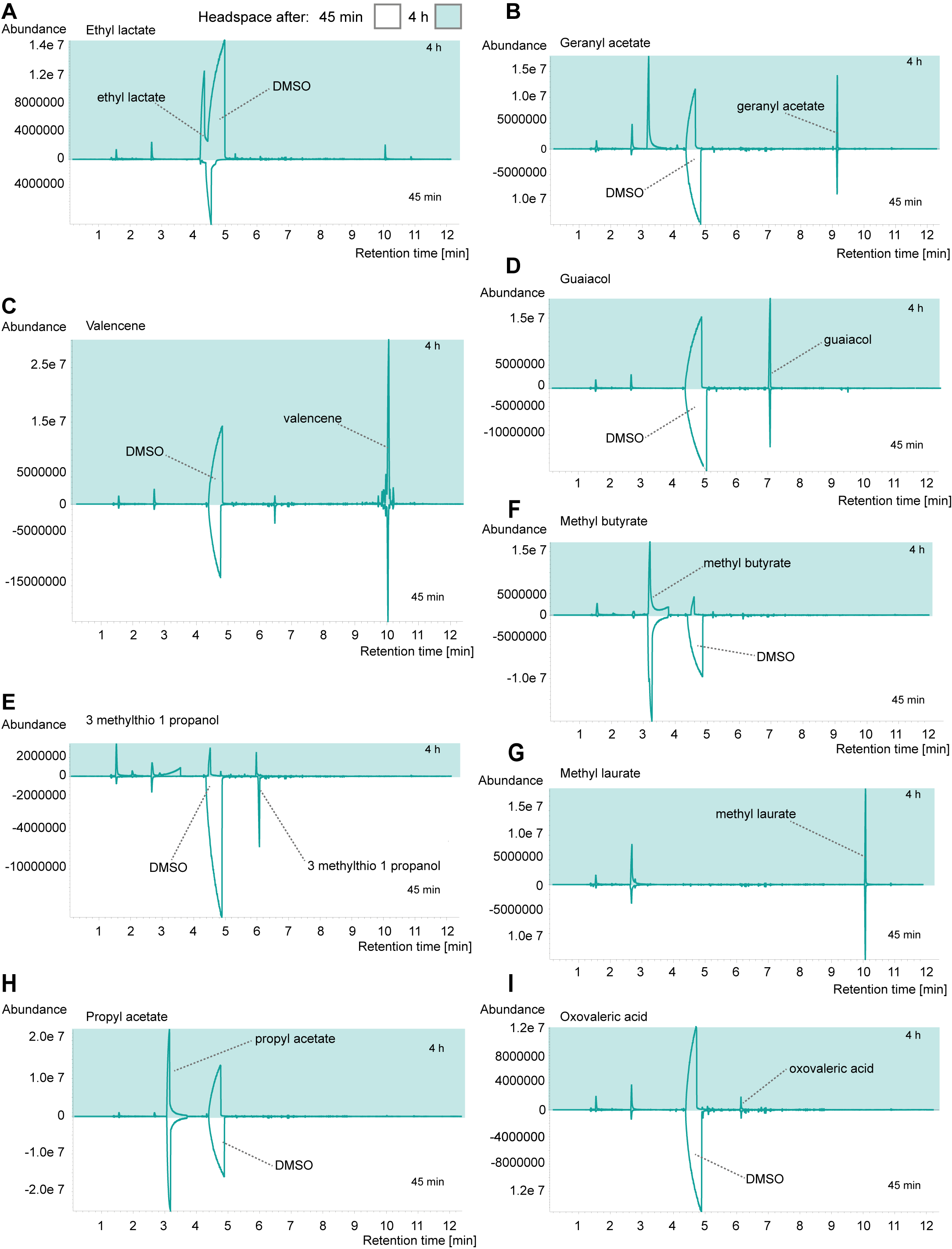

Supplement: FIGURE S3 — Odorant integrity remains unchanged and odorant abundance is stable during long-time exposure of DREAM treatment (A–I) GC-MS profiles of the headspace in fly rearing vials without fly food and flies after 45 min (white background) and 4 h (light green background). In each panel, the odorant used in the DREAM treatment series is highlighted together with its solvent dimethyl sulfoxide (DMSO), with the exception of methyl laurate in which case the solvent methanol was already completely evaporated before the first headspace was collected (G). [file Image_3.TIF]

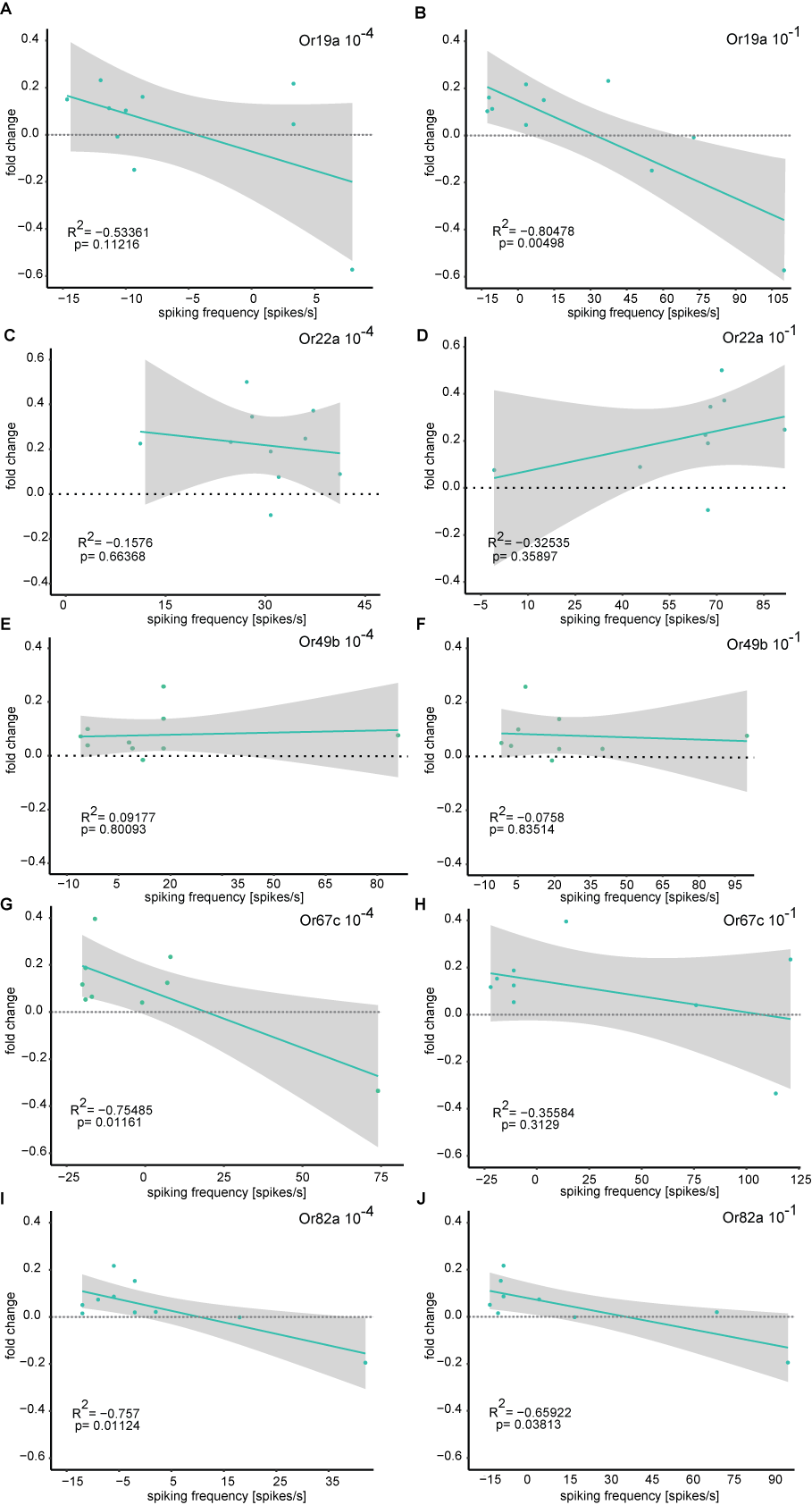

Supplement: FIGURE S4 — Alterations in chemosensory receptor mRNA levels upon odorant treatment cannot generally be used to predict the nature of ligand-receptor interactions. (A–J) Correlation of up- and downregulation events in mRNA levels of Or19a, Or22a, Or49b, Or67c, and Or82a against olfactory sensory neuron spiking frequency upon odorant exposure and odorant stimulation at a dilution of 10−4 or 10−1, respectively. Data points represent the individual odorants tested in the DREAM experiments. In each panel, the gray area indicates a confidence interval of 95%. [file Image_4.TIF]

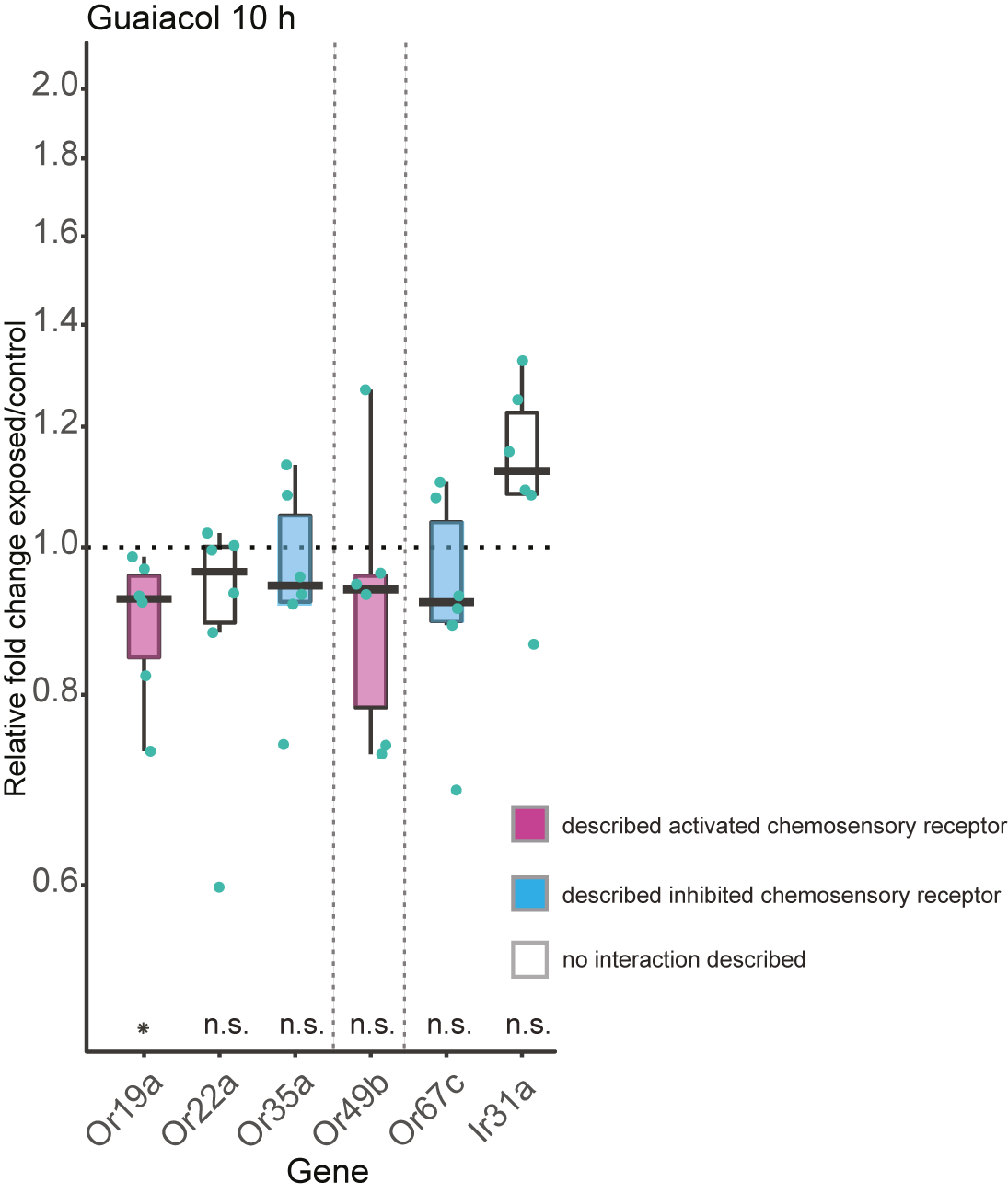

Supplement: FIGURE S5 — A 10 h odorant exposure to guaiacol indicates a trend to mRNA level downregulation of the best ligand–receptor pairing which was previously unresponsive after an exposure duration of 5 h. Evaluation of chemosensory receptor mRNA levels after exposure to 5% v/v guaiacol for 10 h with qPCR. Each data point represents one biological replicate and consists of a pool of RNA from 50 Drosophila melanogaster heads with a mixed sex ratio of 1:1. The total amount of biological replicates equaled six. On the x-axis results from a One sample t-test against 1 are shown. Asterisks indicate significant differences (n.s. P > 0.05; ∗P < 0.05). Highlighted between the two-dotted lines is the best described chemosensory receptor for guaiacol. A cyan box indicates a described and measured inhibitory ligand–receptor interaction while a magenta box refers to a described and measured excitatory ligand-receptor interaction. [file Image_5.TIF]
