# Supplementary material for: Evaluation of the DREAM Technique for a High-Throughput Deorphanization of Chemosensory Receptors in Drosophila
Source: Front Mol Neurosci. 2018 Oct 9;11:366. doi: 10.3389/fnmol.2018.00366 (PMC6189519; doi:10.3389/fnmol.2018.00366)
Supplement: TABLE S1 — Primers used in qPCR experiments. Asterisks indicate primers identical to those from von der Weid et al. (2015). [file Table_1.DOCX]

Table S1 Primers used in qPCR experiments. Asterisks indicate primers identical to those from von der Weid et al. 2015.

| Primer | GenBank accession number | Sequence |
| --- | --- | --- |
| Cam F * | NM_078986.3 | AGCTGACAGAGGAACAGATCG |
| Cam REV * | NM_078986.3 | GCATAACTGTGCCCAACTCC |
| ORCO F * | NM_079511.5 | AAGACCTTCGTCCAGATCGT |
| ORCO REV * | NM_079511.5 | CACCCAGAACCGAAGCAAAC |
| EF1b F * | NM_080069.4 | GTCATCGAGGACGACAAGGT |
| EF1b REV * | NM_080069.4 | TCTTGTTGAAGGCAGCAATG |
| Or19a F * | NC_004354.4 | GATCATTTTGCGCCTCTTCAAGTC |
| Or19a REV * | NC_004354.4 | CCGCCGTGTAGCAGAGAAG |
| Or22a F | NT_033779.5 | TTTTCTGATGACGGAGGCCATCT |
| Or22a REV | NT_033779.5 | GCGTCAACATAGTCCAATAGCAATC |
| Or35a F | NT_033779.5 | GCCTTTACTACGGATTCCCTC |
| Or35a REV | NT_033779.5 | GCTTGCTGTTCATCTCAATGGC |
| Or47a F | NT_033778.4 | GGAGAACCTGAAGACGGAGAGT |
| Or47a REV | NT_033778.4 | GTGCGAACAATCGATGAGAAGGC |
| Or47b F | NT_033778.4 | CCTCGCAGCACAACCTAATGAG |
| Or47b REV | NT_033778.4 | CCAATTTGATAATGTGCTGGTGGAAG |
| Or49b F * | NT_033778.4 | CCTACGAAACGGAGTGGTTC |
| Or49 REV * | NT_033778.4 | TTGCCCAGTAGTATTGCAGC |
| Or67c F * | NT_037436.4 | AAGTGGTTTCAGTGCAGCAA |
| Or67c REV * | NT_037436.4 | CGACATGCTGATGACCTTCATA |
| Or82a F * | NT_033777.3 | CAATTGGCATCTGGCTTCTCC |
| Or82 REV * | NT_033777.3 | TCGACAGATCCCAACGAAATTAG |
| Ir31a F | NT_033779.5 | GTCTTATTCCGAGAGAGTCAAAATCG |
| Ir31a REV | NT_033779.5 | ACCGGAGACTTCACGCAGATC |
