# Supplementary material for: Evaluation of the DREAM Technique for a High-Throughput Deorphanization of Chemosensory Receptors in Drosophila
Source: Front Mol Neurosci. 2018 Oct 9;11:366. doi: 10.3389/fnmol.2018.00366 (PMC6189519; doi:10.3389/fnmol.2018.00366)
Supplement: TABLE S2 related to Figure 2 — Numerical values of Single Sensillum Recording measurements in spikes/s for odorants used in the DREAM treatment at a dilution of 10−4 and 10−1. [file Table_2.DOCX]

Table S2, related to Fig. 2 Numerical values of Single sensillum recording measurements in spikes/s for odorants used in the DREAM treatment at a dilution of 10^-4^ and 10^-1^

| Odorant/chemosensory receptor | Or19a | Or22a | Or35a | Or47a | Or47b | Or49b | Or67c | Or82a | Ir31a |
| --- | --- | --- | --- | --- | --- | --- | --- | --- | --- |
| Valencene 10^-4^ | 8 | 41 | -4 | -10 | 5 | -4 | -1 | -2 | -6 |
| Methyl butyrate 10^-4^ | -9 | 36 | 3 | -17 | 13 | 8 | -20 | -2 | -4 |
| Methyl-1-propanol 10^-4^ | -15 | 28 | 18 | 11 | 9 | 9 | -16 | 18 | -7 |
| Propyl acetate 10^-4^ | -11 | 27 | -2 | 56 | 5 | -4 | -26 | -12 | -4 |
| Methyl laurate 10^-4^ | 3 | 31 | -3 | 12 | 15 | 18 | -19 | -6 | 1 |
| Guaiacol 10^-4^ | -11 | 32 | -1 | -12 | 16 | 86 | -17 | 2 | -10 |
| Ethyl lactate 10^-4^ | -10 | 37 | -7 | -19 | -1 | 18 | 74 | -9 | -4 |
| Geranyl acetate 10^-4^ | -9 | 11 | -5 | -7 | 1 | -6 | 8 | 42 | -4 |
| 2-Oxovaleric acid 10^-4^ | -12 | 25 | 6 | -17 | 15 | 12 | 7 | -12 | -2 |
| Valencene 10^-1^ | 110 | 46 | 17 | 11 | 9 | 2 | 76 | 70 | 3 |
| Methyl butyrate 10^-1^ | -12 | 92 | 3 | -13 | 1 | -2 | -22 | -9 | 3 |
| Methyl-1-propanol 10^-1^ | 11 | 68 | 109 | 19 | 5 | 40 | 14 | 18 | -8 |
| Propyl acetate 10^-1^ | -11 | 72 | -1 | 105 | 7 | 5 | -19 | -10 | 2 |
| Methyl laurate 10^-1^ | 3 | 67 | -1 | 3 | 63 | 22 | -11 | -8 | -4 |
| Guaiacol 10^-1^ | 73 | -1 | -9 | -35 | 5 | 100 | -36 | -42 | -6 |
| Ethyl lactate 10^-1^ | -13 | 72 | 23 | -20 | -1 | 8 | 114 | 5 | -3 |
| Geranyl acetate 10^-1^ | 55 | 66 | -14 | 3 | 1 | -40 | 121 | 96 | -9 |
| 2-Oxovaleric acid 10^-1^ | 37 | 130 | 1 | -16 | 15 | 19 | -11 | -13 | 53 |
