# Supplementary material for: Evaluation of the DREAM Technique for a High-Throughput Deorphanization of Chemosensory Receptors in Drosophila
Source: Front Mol Neurosci. 2018 Oct 9;11:366. doi: 10.3389/fnmol.2018.00366 (PMC6189519; doi:10.3389/fnmol.2018.00366)
Supplement: TABLE S3 — Results of a correlation analysis between up- and downregulation in receptor mRNA levels and spiking frequency of olfactory sensory neurons expressing indicated receptor upon stimulation with odorants tested in the DREAM experiments. [file Table_3.DOCX]

| Chemosensory receptor/ SSR data- dilution series | Correlation coefficient | p-value | Significance level |
| --- | --- | --- | --- |
| Or19a 10^-4^ | -0.53361 | 0.11216 | n.s. |
| Or19a 10^-1^ | -0.80478 | 0.00498 | ** |
| Or22a 10^-4^ | -0.1576 | 0.66368 | n.s. |
| Or22a 10^-1^ | 0.32535 | 0.35897 | n.s. |
| Or35a 10^-4^ | 0.49894 | 0.14209 | n.s. |
| Or35a 10^-1^ | 0.56733 | 0.08718 | n.s. |
| Or47a 10^-4^ | -0.2998 | 0.40002 | n.s. |
| Or47a 10^-1^ | -0.52137 | 0.12223 | n.s. |
| Or47b 10^-4^ | 0.46007 | 0.02127 | n.s. |
| Or47b 10^-1^ | -0.8589 | 0.08261 | n.s. |
| OR49b 10^-4^ | 0.09177 | 0.80093 | n.s. |
| Or49b 10^-1^ | -0.08589 | 0.83514 | n.s. |
| Or67c 10^-4^ | -0.75485 | 0.01161 | * |
| Or67c 10^-1^ | -0.35584 | 0.3129 | n.s. |
| Or82a 10^-4^ | -0.7570 | 0.01124 | * |
| Or82a 10^-1^ | -0.65922 | 0.03813 | * |
| Ir31a 10^-4^ | -0.33111 | 0.35003 | n.s. |
| Ir31a 10^-1^ | -0.34603 | 0.32736 | n.s. |

Table S3 Results of a correlation analysis between up- and downregulation in receptor mRNA levels and spiking frequency of olfactory sensory neurons expressing indicated receptor upon stimulation with odorants tested in the DREAM experiments.
