# Supplementary material for: Evaluation of the DREAM Technique for a High-Throughput Deorphanization of Chemosensory Receptors in Drosophila
Source: Front Mol Neurosci. 2018 Oct 9;11:366. doi: 10.3389/fnmol.2018.00366 (PMC6189519; doi:10.3389/fnmol.2018.00366)
Supplement: TABLE S4 — Survival rate of 50 D. melanogaster flies exposed to dimethyl sulfoxide only (DMSO; control) or 5% v/v of oxovaleric acid in DMSO after 24 h in the DREAM experimental setup. [file Table_4.DOCX]

|  | Survival after 24 h [%] | |
| --- | --- | --- |
| Vial Nr. | **Control** | **Oxovaleric acid** |
| 1 | 100 | 100 |
| 2 | 100 | 100 |
| 3 | 100 | 100 |
| 4 | 99 | 100 |
| 5 | 100 | 100 |
| 6 | 100 | 100 |

**Table S4** Survival rate of 50 *D. melanogaster* flies exposed to dimethyl sulfoxide only (DMSO; control) or 5 % v/v of oxovaleric acid in DMSO after 24 h in the DREAM experimental setup.
