# Supplementary material for: Evaluation of the DREAM Technique for a High-Throughput Deorphanization of Chemosensory Receptors in Drosophila
Source: Front Mol Neurosci. 2018 Oct 9;11:366. doi: 10.3389/fnmol.2018.00366 (PMC6189519; doi:10.3389/fnmol.2018.00366)
Supplement: TABLE S5 — Differences between Cq (threshold cycle) of control and treatment samples for the two reference genes CAM and ORCO. [file Table_5.DOCX]

| Odorant | Reference gene ΔCq | | | | | | | | | | | | | | | | |
| --- | --- | --- | --- | --- | --- | --- | --- | --- | --- | --- | --- | --- | --- | --- | --- | --- | --- |
| Ethyl lactate | CAM | 0.11 | 0.13 | 0.42 | 0.02 | 0.13 | 0.92 | 0.22 | 0.18 | 0.17 | 0.38 | 0.19 | 0.07 | 0.19 | 0.48 | 0.25 | 0.32 |
|  | ORCO | 0.83 | 0.86 | 0.32 | 0.15 | 0.92 | 2.43 | 0.12 | 0.22 | 0.75 | 0.87 | 0.20 | 0.43 | 1.00 | 1.90 | 0.23 | 0.13 |
| Geranyl acetate | CAM | 0.14 | 0.39 | 0.55 | 0.40 | -0.68 | 0.30 | 0.04 | 0.36 | 0.17 | 0.38 | 0.19 | 0.07 | 0.19 | 0.48 | 0.25 | 0.32 |
|  | ORCO | 0.64 | 0.61 | 0.80 | 0.74 | -0.04 | 0.72 | 0.28 | 0.38 | 0.75 | 0.87 | 0.20 | 0.43 | 1.00 | 1.90 | 0.23 | 0.13 |
| Valencene | CAM | 0.14 | -0.03 | -0.08 | -0.17 | 0.28 | 0.22 | 0.34 | 0.31 | 0.13 | 0.10 | 0.14 | -0.09 | 0.01 | 0.36 | 0.09 | 0.60 |
|  | ORCO | -0.21 | -0.07 | 0.14 | -0.05 | 0.01 | 0.18 | 0.45 | 0.45 | 0.02 | 0.06 | 0.35 | -0.07 | 0.01 | 0.08 | 0.66 | 0.39 |
| Guaiacol | CAM | 0.53 | 0.22 | 0.40 | 0.43 | 0.41 | 0.08 | -0.11 | -0.18 | 0.37 | 0.43 | 0.34 | 0.50 | 0.26 | 0.12 | -0.12 | 0.39 |
|  | ORCO | 0.51 | 1.13 | 0.23 | 0.78 | 0.45 | 0.21 | 0.29 | 0.01 | 0.22 | 0.76 | 0.45 | 0.42 | 0.30 | -0.15 | 0.29 | 0.62 |
| 3-Methylthio-1-propanol | CAM | 0.35 | 0.14 | 0.15 | 0.86 | 0.72 | 0.03 | 0.65 | 0.32 | 0.86 | 0.44 | 0.35 | 0.22 | 0.15 | 0.37 | 0.61 | 0.45 |
|  | ORCO | 0.53 | 0.38 | 0.49 | 1.21 | 0.71 | 0.21 | 1.37 | 1.36 | 0.98 | 0.59 | 0.14 | 0.38 | 0.50 | 0.61 | 1.20 | 0.79 |
| Methyl butyrate | CAM | 0.51 | 0.51 | 0.35 | 0.37 | 0.12 | 0.08 | 0.35 | 0.37 | 0.58 | 0.14 | 0.52 | 0.54 | 0.07 | 0.19 | 0.68 | 0.41 |
|  | ORCO | 0.21 | 0.71 | 0.41 | 0.73 | 0.24 | 0.67 | 0.41 | 0.73 | 0.02 | 0.54 | 0.46 | 0.79 | 0.26 | 0.74 | 0.87 | 0.79 |
| Methyl laurate | CAM | -0.11 | 0.36 | 0.78 | -0.04 | -0.18 | 2.03 | -0.84 | -0.05 | -0.37 | 0.17 | 0.85 | 0.12 | -0.13 | 2.20 | 0.90 | 0.38 |
|  | ORCO | -0.02 | 0.15 | 1.34 | 0.66 | -0.18 | 2.12 | -0.42 | 0.01 | 1.33 | 0.00 | -0.13 | 2.09 | -0.56 | 0.15 | 0.21 | 0.16 |
| Oxovaleric acid | CAM | -0.19 | 0.09 | 0.41 | 0.24 | 0.69 | -0.04 | 0.34 | 0.28 | -0.25 | -0.13 | 0.51 | 0.63 | 0.52 | 0.10 | 0.33 | 0.58 |
|  | ORCO | -0.19 | 0.37 | 0.79 | 0.62 | 0.99 | 0.09 | 0.77 | 0.82 | -0.14 | 0.57 | 0.62 | 0.81 | 0.69 | 0.18 | 0.60 | 0.94 |

Table S5 Differences between Cq (threshold cycle) of control and treatment samples for the two reference genes CAM and ORCO.
